# Evaluation of Peripheral Electrostimulation Thresholds in Human Model for Uniform Magnetic Field Exposure

**DOI:** 10.3390/ijerph19010390

**Published:** 2021-12-30

**Authors:** Yosuke Suzuki, Jose Gomez-Tames, Yinliang Diao, Akimasa Hirata

**Affiliations:** 1 Department of Electrical and Mechanical Engineering, Nagoya Institute of Technology, Nagoya 466-8555, Japan; y.suzuki.694@nitech.jp (Y.S.); ahirata@nitech.ac.jp (A.H.); 2 Center of Biomedical Physics and Information Technology, Nagoya Institute of Technology, Nagoya 466-8555, Japan; 3 College of Electronic Engineering, South China Agricultural University, Guangzhou 510642, China; diaoyinliang@ieee.org; 4 Frontier Research Institute for Information Science, Nagoya Institute of Technology, Nagoya 466-8555, Japan

**Keywords:** dosimetry, human safety, nerve model, multi-scale, standardization, uniform exposure, occupational and public protection

## Abstract

The external field strength according to the international guidelines and standards for human protection are derived to prevent peripheral nerve system pain at frequencies from 300–750 Hz to 1 MHz. In this frequency range, the stimulation is attributable to axon electrostimulation. One limitation in the current international guidelines is the lack of respective stimulation thresholds in the brain and peripheral nervous system from in vivo human measurements over a wide frequency range. This study investigates peripheral stimulation thresholds using a multi-scale computation based on a human anatomical model for uniform exposure. The nerve parameters are first adjusted from the measured data to fit the peripheral nerve in the trunk. From the parameters, the external magnetic field strength to stimulate the nerve was estimated. Here, the conservativeness of protection limits of the international guidelines and standards for peripheral stimulation was confirmed. The results showed a margin factor of 4–6 and 10–24 times between internal and external protection limits of Institute of Electrical and Electronics Engineers standard (IEEE C95.1) and International Commission on Non-Ionizing Radiation Protection guidelines, with the computed pain thresholds.

## 1. Introduction

Limits or restrictions for non-ionizing electromagnetic field exposures were developed and published by the International Commission on Non-Ionizing Radiation Protection (ICNIRP) [1,2] and the Institute of Electrical and Electronics Engineers International Committee on Electromagnetic Safety (IEEE ICES) Technical Committee 95 [3,4]. These guidelines and standards are set to protect from stimulation and thermal effects, which are the lowest exposure levels that can cause adverse health effects, therefore, protecting against any other effects. In addition, these guidelines do not address product safety issues, which are intended to limit EMF emissions from specific devices under specified test conditions [5,6].

The standard/guidelines describe that the dominant effect for instantaneous exposure is electrostimulation up to 100 kHz (5–10 MHz for brief pulse exposures). The thermal effect is dominant at frequencies higher than 100 kHz for continuous exposure due to the absorption of energy from electromagnetic fields. This work focuses on the electrostimulation effect at intermediate frequencies (300 Hz to 1 MHz).

The physical quantity (surrogate) of electrostimulation is specified as an internal (in situ) electric field in both guidelines and standards. IEEE protects against adverse peripheral nerve stimulation for frequencies >750 Hz for all environments. ICNIRP addresses peripheral nerve stimulation for frequencies >300 Hz (general public) and >400 Hz (occupational exposure). Above these transition frequencies and lower than 100 kHz, the pain due to peripheral stimulation (axon firing) becomes dominant. Thus, the limit has been set to prevent stimulation attributable to this axon firing.

The limits are named basic restriction (BR) and dosimetric reference limit (DRL) in ICNIRP and IEEE, respectively. In the IEEE standard, the DRLs for the peripheral nervous system were derived from an internal electric field threshold estimated from a 20 μm fiber model (SENN: spatially extended nonlinear node) model excited by a uniform electric field [7] that were consistent with experimental thresholds [8,9,10]. Reduction factors were applied to the above threshold to obtain the DRLs. In the ICNIRP guidelines for the peripheral nervous system, the internal electric field threshold was based on experimental measurements, to which the reduction factor is applied to obtain the BRs.

In the IEEE C95.6 standards, which was revised as IEEE C95.1 standard in 2019, an ellipse is used to relate an external magnetic field to an internal electric field. The ICNIRP 2010 guidelines used anatomical model computation to derive the relationship between external magnetic field strength and the internal electric field.

Due to the lack of relation between internal and external field strength and nerve activation, the internal and external field strength in the guidelines and standard is thus derived conservatively. Only a few experimental studies are worth replicating the relationship between the spatially non-uniform external field strength and the pain [8,9,10]. Another limitation is that the experimental threshold evaluation is difficult across the frequency range, and in vivo measurement of the electric field in millimeter resolution cannot be conducted. For these reasons, the simulation method is commonly used [11]. In addition, to understand the stimulation from the external field, the multi-scale simulation, i.e., the electromagnetics and neuron model, is needed, as is listed in the research agenda of the IEEE ICES [12]. In addition, ICNIRP recommended that excitation modeling and threshold assessment be conducted to improve the accuracy of restrictions [11]. These recommendations and computational advances in incorporating neurons into realistic human models [13,14,15,16,17,18,19] motivate revisiting the current protection limits of the standard and guidelines and provide rationality of the applied reduction factors as safety margins. A few studies have evaluated the threshold for central nervous system stimulation considering axonal stimulation indicating conservativeness with respect to the current guidelines/standard presented at low frequency [20,21]. However, systematic analysis of the peripheral nervous system has not been conducted for human safety.

This study estimates the relationship between the internal electric field and the external magnetic field thresholds based on numerical dosimetry analysis to discuss the protection limits to the electrostimulation effect at intermediate frequencies. First, we used an electromagnetic field analysis integrated with an axonal model derived by replicating experimental measurements. Then, the activation model for peripheral threshold is derived computationally at a frequency of 300 Hz–1 MHz. Then, the conservativeness of protection limits in the international guidelines/standards is discussed.

## 2. Materials and Methods

### 2.1. Human Body Model

An anatomical model of a Japanese adult man (TARO) [22] was used to model a volume conductor. The height and weight of TARO were 173.2 cm and 65 kg, respectively. The original TARO model consisted of more than 40 million voxels with a 2 mm resolution. The number of tissues considered in this model was 51, including skin, muscle, bone, and heart. The resolution of the model was converted into 1 mm for representing the target tissue in which the nerve was placed with higher resolution. The electrical conductivity of the tissues was determined based on the 4-Cole-Cole model [23] and assigned to each tissue.

### 2.2. Electromagnetic Computational Method

At frequencies up to 10 MHz, biological tissue was assumed not to perturb the external magnetic field. In this frequency range, a magneto-quasistatic approximation applies to the computation of the internal electric field in biological tissues [24]. The applicability of the quasistatic approximation was confirmed in [25] by comparison with full-wave analysis. In addition, the conduction currents were at least one order of magnitude higher than the displacement currents, and, therefore, tissue electrical conductivity was considered, while the permittivity can be neglected [26,27].

The internal electric field was computed by substituting the magnetic vector potential distribution into an electromagnetic solver based on the scalar potential finite difference (SPFD) method. In this method, the body models were discretized with cubic voxels, and simultaneous linear equations were developed for all contacts, with the electric scalar potential as unknown variables [28].

(1) $$\sum_{n=1}^{6}s_{n}\phi_{n}-\left(\sum_{n=1}^{6}s_{n}\right)\phi_{0}=j\omega \sum_{n=1}^{6}{\left(-1\right)}^{n}s_{n}l_{n}A_{0n}$$

where *A_0n_*, *s_n_*, *l_n,_* and *ω* denote the magnetic vector potential of the applied uniform magnetic field, edge conductance derived from the tissue conductivity, the length between the nodes, and the angular frequency, respectively. The SPFD defines the scalar potentials ϕ (unknowns) at each node *n* of a voxel, and a branch current flowing from one node to a neighboring one along the side of a voxel was derived, which includes the vector potential owing to the applied magnetic field. In this study, the scalar potential was computed iteratively via the successive-over-relaxation and multigrid methods [24]. When (1) is solved, the internal electric field ***E*** is calculated as the following: ***E*** = − ∇ϕ − *jω**A***_0_.

This study evaluated the electric field strength calculated using SPFD using the 2 mm cubic averaging method and 5 mm line averaging [29] following the criteria dose for evaluating BR compliance as defined by ICNIRP and IEEE, respectively. ICNIRP recommends determining the internal electric field “as a vector average of the electric field in a small contiguous tissue volume of 2 × 2 × 2 cubic millimeters. For a specific tissue, the 99th percentile value of the electric field was the relevant value to be compared with the BR.” In addition, we considered the criteria for IEEE, “the in situ electric field DRL applies to the rms electric field strength measured in the direction and location providing the maximum in situ electric field vector (vector magnitude) over a 5 mm linear distance.”

### 2.3. Neuronal Activation Computational Models

The effects of the induced electric field on peripheral nerve axons were described in the compartmental form [30]:

(2) $$C_{m,n}\frac{dV_{m,n}}{dt}+I_{ion,n}-\frac{V_{m,n-1}-2V_{m,n}+2V_{m,n+1}}{0.5\left(R_{m,i}+R_{m,n}\right)}=\frac{V_{e,n-1}-2V_{e,n}+2V_{e,n+1}}{0.5\left(R_{m,i}+R_{m,n}\right)}$$

Thus each compartment corresponds to nodes (*n*) or internodes (*i*), forming a myelinated axon fiber that consists of membrane capacitance (*C_m_*) and axial resistances (*R_m,i,_* and *R_m,n_*). The term *V*_m_ denotes the membrane potential along the cable; the quasi-potential *V*_e_ is the line integral of the internal electric field, obtained in the previous section, along the path of the fiber. At the nodes of Ranvier, the ionic membrane current was developed using a conductance-based voltage-gated model of sodium and leakage currents (Chiu–Ritchie–Rogart–Stagg–Sweeney model or CRRSS model) [31]. At the internodes, the membrane current *I_ion,n_* was modeled by the passive conductance multiplied by the membrane potential. The electrical parameters of the CRRSS model are shown in Table 1. Note that the membrane parameters in the CRRSS model were derived from the measurement of rabbit [32]. The threshold value was obtained when the membrane potential was depolarized up to 80 mV in at least four neighboring nodes at successive times [33].

### 2.4. Strength-Duration (S-D) Curve

The strength-duration curve or S-D curve shows the relationship between the stimulation threshold and pulse duration. The S-D curve can be expressed by 2 quantities: rheobase and chronaxie. The former was the minimum current intensity that elicits an activation after a long duration; the latter was the pulse duration corresponding to a stimulation current that was twice the rheobase value. The S-D curve relationship can be expressed by the following equation:

(3) I=b(1+C/w)

where *I* is the input current, *w* is the pulse width of the stimulation, *b* is the rheobase, and *C* is the chronaxie. These parameters describe the excitability characteristics of different nerve fibers and are usually reported in experimental measurements.

### 2.5. Experimental Stimulation Thresholds

The compartmental model parameters in Equation (2) were adjusted based on an experimental S-D curve derived from the reported magnetic field simulation of the torso using a gradient coil in another study [34]. The experimental S-D curve in [34] was obtained using a scale from 0 to 10 according to the reported response by changing the stimulation strength at different durations (50–1000 μs). The minimum and maximum in the scale corresponded to no feeling and intolerable. To match the experimental and computational S-D curves, parameters of *C*, *G_l_*, *G_na_* were used to fit the rheobase and chronaxie values [18,35]. Additionally, the diameter of the peripheral model was set to 20 μm as used in the IEEE standard.

In this study, we considered the uncomfortable excitation threshold (scale 5) as an adverse reaction for comparison with the protection limits in addition to the reported perception threshold (scale one) [9,34]. The reported rheobase values derived from the experimental strength–duration curve were 28.3 T/s and 18.8 T/s for uncomfortable and perceptual responses, respectively. The ratio between uncomfortable and perceptual rheobases (1.5) was according to the multiplier factor of 1.45 between painful stimulation and perception in IEEE standard. The measured chronaxie was 360 μs [34].

The corresponding rheobase value of the internal electric field (*E_th_*) was derived from the following equation [9]:

(4) $$E_{th}=E_{sim}\frac{{\left(\frac{dB}{dt}\right)}_{th}}{{\left(\frac{dB}{dt}\right)}_{sim}}$$

where (*dB/dt)_th_* is the measured value of the rheobase (external), (*dB/dt)_sim_* and *E_sim_* were the external magnetic field and internal field obtained by dosimetry analysis simulating the gradient coil [9]. Using Equation (4), the rheobase expressed as internal electric field values were 4.8 V/m and 7.3 V/m for perceptual and uncomfortable excitation thresholds, respectively.

**Figure 1:**
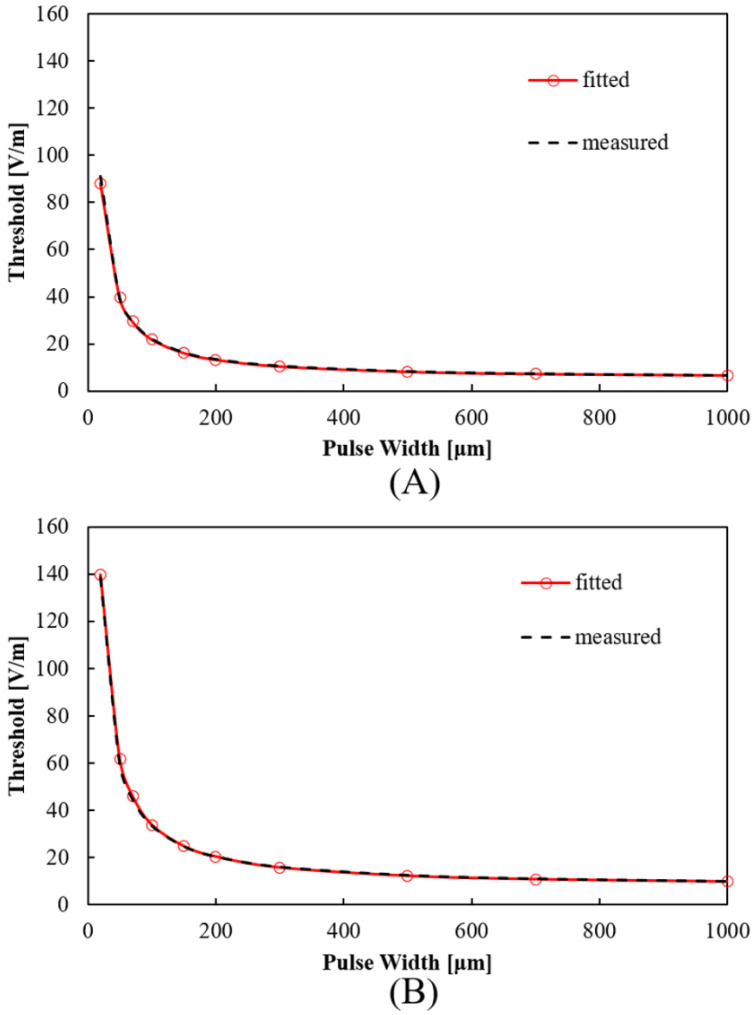
Experimental and computed S-D curves of (**A**) perceptual thresholds and (**B**) uncomfortable thresholds. Experimental results are presented with permission from [34].

For the parameter fitting using the S-D curve in Section 3.1 (see Figure 1), a bipolar square wave simulated the same exposure waveform in the experimental condition [34]. The pulse widths were 20, 50, 100, 150, 200, 300, 500, 700, and 1000 μs, and the pulse interval was set to 300 μs.

### 2.6. Computational Exposure Scenarios

**Figure 2:**
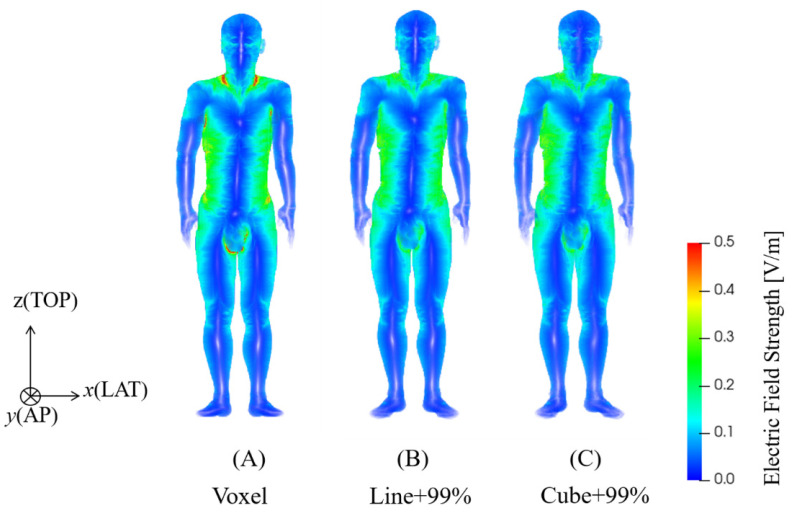
The internal electric field strength in TARO model using (**A**) no averaging (voxel value), (**B**) 5 mm linear averaging, and (**C**) 2 mm cubic averaging for uniform magnetic field exposure intensity of 0.3 mT at 1 kHz.

The body model was exposed to a uniform magnetic field from the front-to-back direction as the one producing the highest internal electric fields. In Section 3.2 and Section 3.3, the frequency was set to 1 kHz to investigate the internal electric field distribution and threshold dependence on nerve orientation (see Figure 2). The differences in the internal electric distribution caused by frequency were marginal, considering the linear variation with frequency.

**Figure 3:**
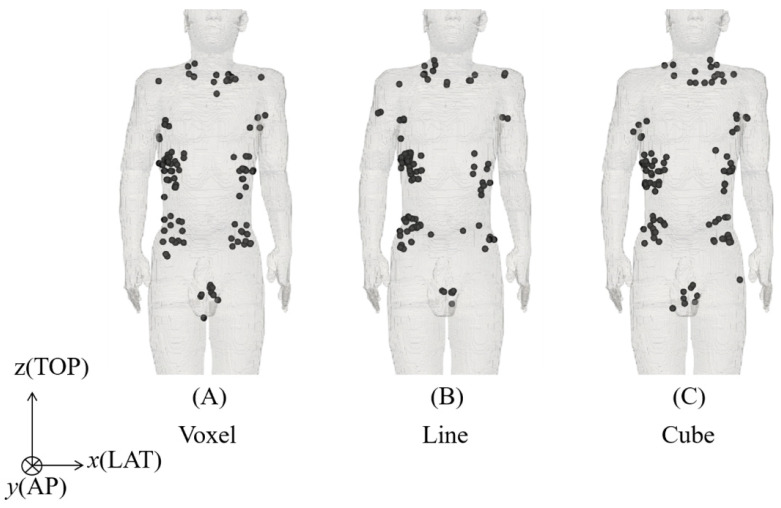
Distribution of the positions of the highest internal electric fields (**A**) voxel and (**B**) 5 mm linear averaging, and (**C**) 2 mm cubic averaging (100 positions are shown for illustration).

**Figure 4:**
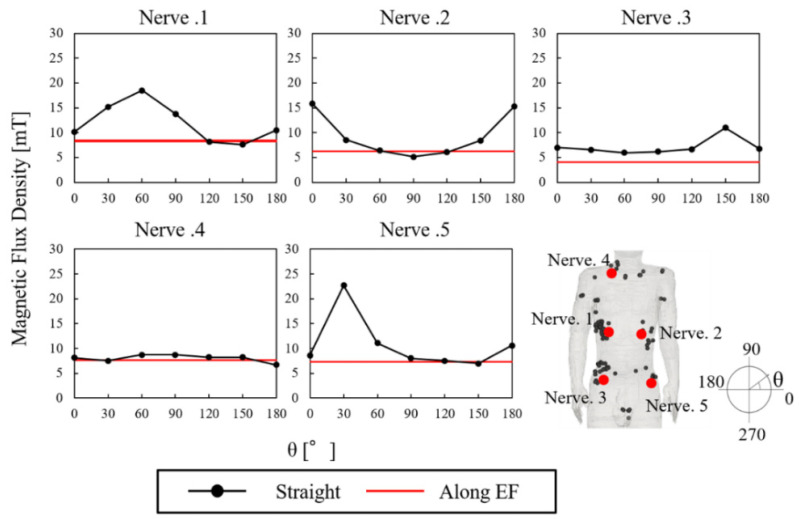
External threshold dependence on straight nerve orientation and nerve curved along the electric field direction.

**Figure 5:**
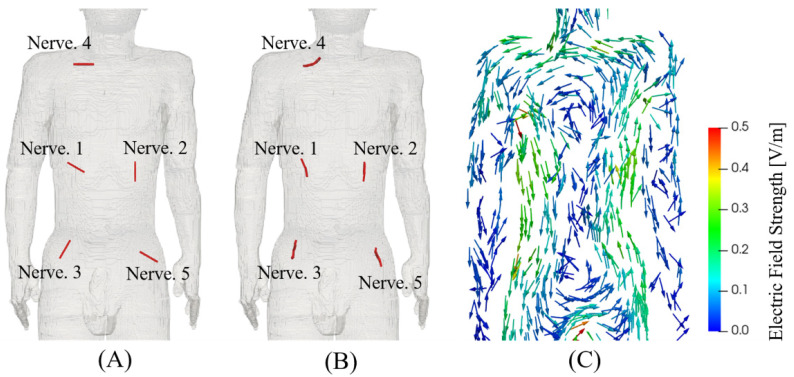
Orientations of the nerves with minimum threshold using (**A**) straight and (**B**) curved nerve. (**C**) The distribution of the electric field vector.

**Figure 6:**
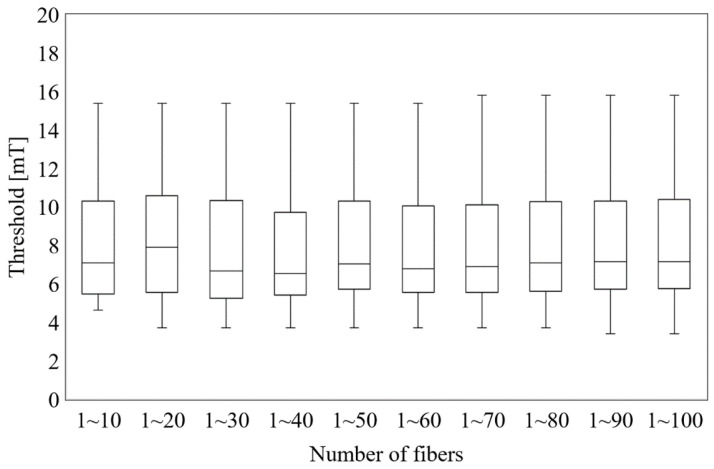
Threshold distribution of curved fibers centered on the top electric field values.

In the standard and guidelines, the nerve was placed in the subcutaneous fat tissue and skin as a surrogate for peripheral nerve tissue. The fibers were centered in positions corresponding to the top electric fields strength values (see Figure 3 and Figure 4), and 2 types of orientations were considered [36]. One was straight fibers with different directions (0° to 180° with steps of 30°, anticlockwise), and the other was fibers bent along the electric field (see Figure 5). Moreover, we considered the thresholds distribution considering different numbers of fibers (10 to 100), as shown in Figure 6. These thresholds were obtained using fibers centered on the top electric fields and oriented along the electric field (see Figure 6).

To assess the protection limits in the international standard/guidelines in Section 3.4, the frequency was set from 300 Hz to 1 MHz. In addition, the computed external stimulation threshold was compared with the external field strength prescribed in the ICNIRP (reference levels for occupational exposure) and IEEE (exposure reference levels for head and torso in restricted environments).

In addition, we considered small fibers related to pain and nerves related to proprioception. A validated myelinated small fiber (Aδ) for pain thresholds of 5 μm was placed in the subcutaneous fat tissue [18]. In addition, we assigned the same myelinated nerve (A-fiber type) with parameters adjusted to 20 μm considering the thickness of Aα-fibers for proprioception in muscle tissue [37,38]. These were included to assess the results’ conservativeness and the nerve model developed in this study.

## 3. Results

### 3.1. S-D Curve Response of Nerve Model

The parameters in the nerve activation model were adjusted to estimate the external magnetic field for the stimulation threshold based on S-D curve experiments during magnetic exposure. The rheobase values of perceptual (4.8 V/m) and uncomfortable (7.3 V/m) were derived from dosimetry analysis in [9] based on experimental results in [34]. We modified the electrical parameters of the CRRSS model (*C*, *G*_na_, and *G*_l_) to fit the computed S-D curve to the experimental. The S-D curve obtained from the fitted nerve activation model is shown in Figure 1 for the estimated parameters in Table 2. The difference between experiment and computed values was up to 10% for rheobase and chronaxie values, considering different nerve positions for the top electric field values (see next section).

### 3.2. Distribution of Hotspots of Internal Electric Field

Figure 2A–C show the original (voxel data), 5 mm line-averaged, and 2 mm cube-averaged internal electric field strength in a human model exposed to a uniform field at 1 kHz. High internal electric fields were mitigated around underarms, crotch, and part of the neck based on the two averaging methods. Figure 3 presents the distribution of the positions with the top internal electric field values on the subcutaneous fat tissue that shows distinct areas. A high concentration of top electric field points was observed in the lateral parts of the chest, abdomen, and also neck. The percentile internal electric fields and distribution were not significantly different between the 5 mm line-averaged and 2 mm cube-averaged methods. We adopted the linear averaging for selecting the positions of the top-electric fields where the fibers were centered in the following sections.

### 3.3. Electrostimulation Threshold Based on Nerve Orientation

We selected peripheral nerves from the positions with top electric field strength values from the distinct regions in the chest, abdomen, and neck and investigated the threshold dependence on nerve orientation. Figure 4 shows the external threshold dependence on straight nerve oriented at different angles θ and the external threshold when the nerve model was curved along the electric field direction. The variation of the straight nerve threshold with the orientation was significant in most of the positions, while its minimum was comparable to stimulation thresholds of the curved nerves. A larger variability was observed for the curved nerve than the straight nerve (4.2 mT vs. 2.5 mT) in terms of the minimum threshold. Figure 5 illustrates the nerve model orientation with the minimum threshold obtained for straight nerve and curved nerves aligned along the electric field.

Figure 6 shows the threshold variability for a group of nerves. We selected only fibers bent along the electric field as they present the smallest threshold for conservative evaluation. Considering different number of fibers (10–100 fibers) and each fiber centered on the top 100 electric fields positions, the median threshold was calculated for each group. We found that the median threshold converges for a group of fibers larger than 30 (6.8 ± 3.5 mT), in which the effect of few fibers with high thresholds is reduced.

### 3.4. Comparison with Protection Limits

**Figure 7:**
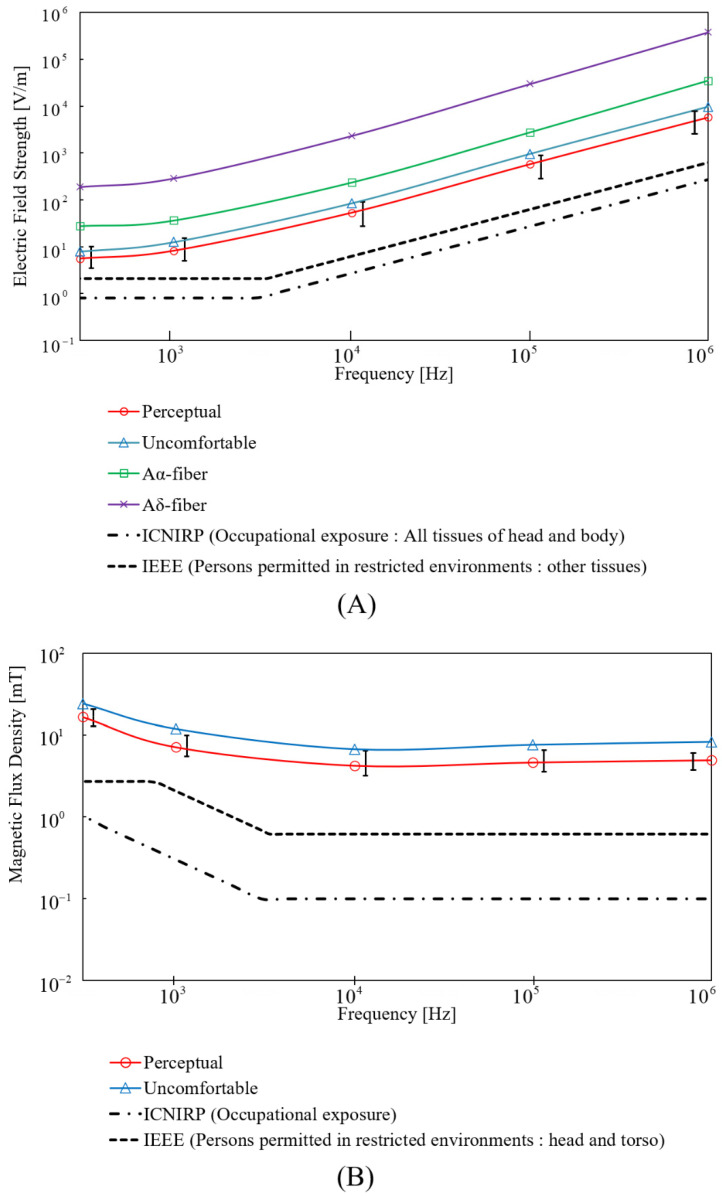
Thresholds of (**A**) internal electric field and (**B**) external magnetic flux density to activate peripheral nerve computed by a multi-scale approach. Comparison with protection levels by ICNIRP (reference levels for occupational exposure) and IEEE (exposure reference levels for head and torso in restricted environments).

Figure 7 shows threshold-frequency curves derived from uniform exposure of the fitted nerve model for perceptual and uncomfortable responses. The fiber corresponding to the median threshold was selected for fitting the space parameter. The computed internal electric field threshold increased with the frequency, from 100 Hz to 1 MHz. Compared with international guidelines/standards, computed internal perceptual thresholds were 6.9- and 2.6-times higher than the limits in the ICNIRP guidelines and then the IEEE standards, respectively. Similarly, computed external perceptual thresholds were 16.8- and 3.4-times higher than the limits in the ICNIRP guidelines and the IEEE standards. For uncomfortable excitation thresholds, computed uncomfortable internal thresholds were 9.9- and 3.8-times higher than the limits in the ICNIRP guidelines and the IEEE standards. External thresholds were conservative, about 24.3-times higher than the ICNIRP guidelines and about 5.7-times higher than the IEEE standards, as shown in Figure 7B. The thresholds for pain myelinated small fibers of 5 μm were about 34 times higher than the perceptual thresholds. Furthermore, the thresholds for large, myelinated fibers in muscle tissue were about 5-times higher than the perceptual thresholds. This result confirms that electromagnetic field strength below the protection levels does not activate the peripheral nerves. In addition, Figure 7 also shows that threshold variability produced by nerves with different bending on different positions of the top electric fields also complies with the conservativeness of the limits.

## 4. Discussion

This work investigated the peripheral nerve stimulation threshold by combined modeling of electromagnetic dosimetry and the nerve activation model verified with an experimental S-D curve for comparison with protection limits in the international guidelines and standard for frequencies of 300 Hz–1 MHz.

Conservative conditions of peripheral stimulation threshold were considered for comparison with the protection levels by the international guidelines and standards. We look into the regions with top-electric fields as a condition for conservative estimation of external thresholds. Two methods (2 mm cubic averaging and 5 mm linear averaging) for spatially averaging the internal electric field in the standard and guidelines were considered to obtain a stable peak estimation [12]. The 2 mm cubic averaging method specified by ICNIRP seems to be more suitable for considering the collective “network” effect of interacting nerve cells. For a specific tissue, the 99th percentile value of the electric field is the relevant value to mitigate maximal values prone to numerical artifacts such as stair-casing errors in voxel models. The 5 mm IEEE linear averaging method was derived from the length of the myelinated nerve exposed in the internal electric field because the nerve excitation seems to be most sensitive to the electric field oriented with the long axis of the nerve fiber. It does not specify a percentile as the uniform isotropic ellipsoidal induction model was adopted for deriving the limits. Although the implementations for the two methods differ, their percentile internal electric fields were not significantly different from one another. Some differences in the averaged electric fields between using the two methods mainly exist on the tissue boundary voxels. This is attributable to the difference in the sets of averaged voxels, as the averaging methods exclude electric field values if the voxels in the averaging volume/line do not belong to the same tissue. In fact, the difference in the averaged electric field for the voxels in common for the two methods is marginal [39]. As shown in Figs. 2 and 3, the distributions of the top electric field positions were relatively consistent in the chest and abdomen areas between the two methods, in line with other studies that have reported no significant difference between the two methods [40]. We also observed a few top-electric fields in the crotch and armpit, although that may appear due to current crossing skin layers that are not applied in current DRLs. In addition, those regions were not reported as the positions of pain in the experimental measurements [34,39]. We then investigated the effect of fiber orientation for fibers centered on the top electric field positions on the chest and abdomen based on the 5 mm linear averaging. We confirmed that the threshold converged to a median value when considering a minimum pool of 30 fiber, and that presented a variability of 50% due to different levels of bending for the fiber aligned along the electric field [36]. Marginal variation of the median thresholds (less than 5%) was observed if the 2 mm cubic averaging method was applied.

The response of the peripheral nerve model was adjusted to reproduce the same experimental S-D curves in terms of the internal electric field of the selected responses. In the standard and guidelines, BR and DRLs are for protection against adverse health effects. In ICNIRP, “the risks come from transient nervous system responses.” In addition, “ICNIRP notes the relatively narrow margin between peripheral nerve perception and pain thresholds.” ICNIRP applies a five-fold safety margin for controlled environments and an additional 2-fold reduction for the public. In the case of IEEE, the dosimetric reference level is based on peripheral nerve stimulation that agrees with a median reaction threshold that corresponds to a detectable response. A multiplier factor of 1.45 is considered for painful stimulation because perception itself is not an adverse effect, in which additional safety factors of three and nine are applied to body parts in a public and controlled environment. Notably, ICNIRP does not explain how the reduction factors are obtained, whereas a rationale is given in the IEEE standard. In this study, the S-D curves corresponded to felt stimulus and uncomfortable responses. The first one is a strict condition. The second is a reasonable comparison to the pain threshold considering the multiplier factor observed in experimental studies (1.3–1.6 times) of pain above perception [41,42,43] and used in the IEEE standard. The mammalian peripheral nerve model was based on the CRRSS model with modified parameters for adjusting the S-D curves. The activation threshold may be adjusted using different combinations of various parameters related to the ionic channels’ morphology and membrane dynamics [38,44]. For simplicity, we adopted a small parameter space for fitting [18], considering that the rheobase depends primarily on the conductance, whereas the chronaxie depends on the capacitance and sodium conductance [35]. The fiber corresponding to the median threshold was selected for fitting the space parameter.

As shown in Figure 7, it is confirmed that the peripheral nerve is not activated by the external electromagnetic field strength specified by ICNIRP and IEEE. Furthermore, it is confirmed that the protection levels are conservatively defined for the discomfort threshold. We also noticed that the threshold for perceptual activation is seven times the protection level in the ICNIRP guidelines, close to the reduction factor of five in the ICNIRP guidelines for “transient nervous system responses.” The threshold of discomfort is four times the protection levels in the IEEE, which is also close to the reduction factor of three defined in IEEE standards for painful stimulation. Indeed, the guidelines and standards incorporate reasonably large margins of safety as they should be especially conservative because the safety factors are applied against perception phenomena (e.g., electrostimulation and behavioral disruption).

Moreover, evaluating additional myelinated nerve fibers models related to pain perception and muscle proprioception yielded even more conservative results. The threshold of the myelinated nerve with a thickness of 20 μm, which is assumed to be the largest nerve in the human body, converged to about 40 V/m at minimum frequency. The electric field threshold presents similar values to the perception threshold on the muscle for magnetic stimulation of the Broca’s at 35–42 V/m as [19]. In addition, we found consistency from rheobase values reported from different exposures, including that used in this work. In the case of contact currents from electrical stimulation, the rheobase value in terms of the internal electric field strength was estimated between 66 to 129 V/m using 1.0 µm for a small fiber during the perception threshold [18]. An adjustment to 20 µm resulted in approximately between 3.3 V/m and 6.4 V/m, considering that the threshold is inversely proportional to the diameter. This agrees with reported induced electric field thresholds in magnetic exposure (2 V/m to 6 V/m) as well [9,34]. Further revision on the agreement of thresholds from different exposures will contribute to the rationality and harmonization of the standard and guidelines.

One limitation is the interpretation of the pain threshold for adverse reactions in experimental results. Although nerve stimulation thresholds based on discomfort and even perception responses did not violate the protection limits, experimental reports are encouraged to investigate this systematically in particular individual variations of electrostimulation threshold. In addition, external electrostimulation thresholds are affected by many factors, such as body size and posture [45]. From internal electric fields obtained in available anatomical models (see [46]), it is possible to estimate a small reduction of the external magnetic field threshold for larger models than TARO model but not significant to violate the protection limits considering the margins estimated in this study (10–24 times higher than the standard/guidelines). It is important to mention also that one significant source of uncertainty is the electrical conductivity (anisotropy, uniformity, age dependency [47,48]), and further studies are necessary in order to improve the accuracy and validity of relevant dosimetry studies. Another limitation is that the experiment employed a gradient coil to investigate the internal electric field threshold. Nevertheless, similar rheobase values obtained from uniform exposure (6.15 V/m) [49] and a coil encircling the forearm (5.9 V/m) [8] and intrinsic characteristics of the nerve threshold are reported. This in part as the gradient coil generates a distribution that resembles uniform exposure in the front-to-back direction. Finally, the external protection limits described in the international exposure guidelines and standard assumes that humans standing in free space are exposed to a uniform field. In addition, there is conservativeness when applying the uniform magnetic field-based criteria for assessment when considering non-uniform exposure in practical applications [50,51].

## 5. Conclusions

This study investigated peripheral stimulation thresholds using a multi-scale computation based on a human anatomical model during uniform magnetic field exposure for the first time in the context of revisiting the protection limits of international standards/guidelines. Computed internal and external field strengths were compared with the limits indicated on IEEE standard and International Commission on Non-Ionizing Radiation Protection (ICNIRP) guidelines for human protection in low frequency (300 Hz to 1 MHz), in which stimulation is attributable to axon electrostimulation. Our results confirmed the conservativeness of protection limits of the international guidelines and standards for peripheral stimulation. The results showed a margin factor of 4–6 and 10–24 times between internal and external protection limits of the international guidelines and standards. The difference between the threshold and the protection limits of the induced electric field was about the same as the reduction factor assumed in the international guidelines and standards. On the other hand, the difference between the threshold and the protection limits of the external magnetic field was more than twice as large as that of the induced electric field. However, it is important to note that the protection limits incorporate reasonably large margins of safety.

## Figures and Tables

**Table 1:** The electrical parameters used in the CRRSS model.

Parameter	Value
Nernst potential for sodium channels (*E_Na_*)	115 mV
Nernst potential for leakage channels (*E_l_*)	−0.01 mV
Capacity of membrane at internode (*C_m,i_*)	28.8 nF
Capacity of membrane at node (*C_m,n_*)	30.2 nF
Internode membrane resistance (*R_m,i_*)	218 kΩ
Nodal membrane resistance (*R_m,n_*)	3.26 kΩ
Myelin conductance (*G_m_*)	26.8 nS
Sodium channel conductance (*G_Na_*)	1445 mS/cm^2^
Leaked channel conductance (*G_l_*)	128 mS/cm^2^

**Table 2:** Adjusted passive parameters of CRRSS for experimental responses.

Parameter	Value	
	Perceptual	Uncomfortable
Capacity of membrane (*C*)	6.0 times	8.5 times
Sodium channel conductance (*G_Na_*)	8.0 times	4.5 times
Leaked channel conductivity (*G_l_*)	0.25 times	0.25 times
